# High Quality *de Novo* Transcriptome Assembly of *Croton tiglium*

**DOI:** 10.3389/fmolb.2018.00062

**Published:** 2018-07-05

**Authors:** Markus Haak, Svenja Vinke, Willy Keller, Julian Droste, Christian Rückert, Jörn Kalinowski, Boas Pucker

**Affiliations:** ^1^Center for Biotechnology, Bielefeld University, Bielefeld, Germany; ^2^Faculty of Biology, Bielefeld University, Bielefeld, Germany; ^3^Department of Biology, Massachusetts Institute of Technology, Cambridge, MA, United States; ^4^Department of Plant Sciences, University of Cambridge, Cambridge, United Kingdom

**Keywords:** *Croton tiglium*, RNA-Seq, Trinity, reciprocal best BLAST hit, annotation transfer, gene expression, tissue-specific, anti-cancer

## Introduction

*Croton tiglium* is one of more than 1,200 different species in the large genus *Croton*, belonging to the family Euphorbiaceae (Kalwij, 2012; The Plant List, 2014). *C. tiglium* can be found in subtropical and tropical regions on both hemispheres (Salatino et al., 2007). This plant was first mentioned in the medical literature over 2,200 years ago in China. The medical relevance is probably due to a huge variety of different secondary metabolites (Pope, 1824). Traditionally utilized as a purgative to treat gastrointestinal and intestinal disorders, as an abortifacient and counterirritant, the commercially available seed oil of *C. tiglium* is nowadays applied in homeopathy and acupuncture (Gläser et al., 1988). The pharmacologic mechanism of the laxative properties of ethanol extracts of *C. tiglium* has been studied on rat intestinal epithelium (Tsai et al., 2004). *C. tiglium* produces various phorbol esters, including substances that were reported to be tumor-promoting (Van Duuren et al., 1963), antileukemic and antimycobacterial (Goel et al., 2007; Salatino et al., 2007), and even candidates for the treatment of HIV (El-Mekkawy et al., 2000). Beside the tumor-promoting factors, some cytotoxic phorbol esters were isolated from plant extracts and evaluated in cell culture assays (Zhang et al., 2013). In contrast to the co-carcinogenic substances, *C. tiglium* was shown to produce a ribonucleoside analog of guanosine with antitumor activity (Kim et al., 1994).

In this work, we present a comprehensive *de novo* transcriptome assembly of *C. tiglium* based on a normalized library to cover a huge variety of transcripts. In addition, tissue-specific transcript libraries were generated to enable differential gene expression analysis between tissues. This will facilitate the identification of candidate genes involved in growth, development, and metabolism of this plant species.

## Materials and methods

### Plant material

Tissue samples from *Croton tiglium* L. were kindly provided by the botanic garden of the Phillipps-Universität Marburg (https://www.uni-marburg.de/botgart). The plants were cultivated in individual buckets in a greenhouse at 18–20°C, a relative humidity of 60–80% and daylight. The substrate consisted of potting soil mixed with sand, lava and clay, fertilized every 2 weeks with WUXAL Top N and WUXAL Super liquid fertilizer. The collected samples were frozen in liquid nitrogen immediately and kept on dry ice until RNA extraction.

### RNA extraction

Total RNA was extracted from frozen tissue samples of young leaves, stem, inflorescence, seeds, and roots. Mortar and pestle were used to grind the material in liquid nitrogen. The Spectrum Plant Total RNA Kit (Sigma Aldrich) was used for RNA extraction according to the supplier's instructions. A DropSense16 (Trinean) was used for quantification and quality control. RNA samples with insufficient purity were purified with the RNA Clean & Concentrator Kit (Zymo Research) prior to library preparation.

### Sequencing library preparation

A pooled total RNA sample from all five tissues was used for the construction of a normalized, stranded library (vertis Biotechnologie AG). In parallel, tissue-specific libraries with an average insert size of 400 bp were prepared according to the Illumina TrueSeq Stranded mRNA Sample Preparation Guide. These libraries represent young leaves, stem, inflorescence, seed, and root.

### Sequencing

Sequencing of the normalized library was performed on two lanes of an Illumina HiSeq1500 in Rapid Run mode generating about 47.4 million 2 × 250 nt paired-end reads. Sequencing of the tissue-specific libraries was performed on the same machine generating between 20 and 44 million 2 × 75 nt paired-end reads per tissue-specific library (Supplementary Table 1).

### Sequencing read data processing

FastQC (Andrews, 2010) was applied to check the quality of all sequencing data. Low quality regions and adapter fragments were removed from the reads via Trimmomatic 0.36 (Bolger et al., 2014). Removal of adapters was performed based on all known Illumina adapter sequences with the options 2:30:10. A sliding window of the length 4 was used to clip reads once the average PHRED score dropped below 15. Reads below the length cutoff of 100 nt were discarded. Pairs with only one surviving read were dropped after trimming.

### *De novo* transcriptome assembly

Trinity v2.4.0 (Grabherr et al., 2011; Haas et al., 2013) was applied with different k-mer sizes and the stranded flag for the *de novo* transcriptome assembly based on all 2 × 250 nt paired-end reads of the normalized library (SRR6239853). Finally, 24 was identified as the best k-mer size based on comparison of the results by assembly size, number of contigs, assembly continuity, and recovered benchmarking genes. The minimal length of contigs to report was set to 200 nt.

Assembly completeness was investigated by computing assemblies for subsets of the data. Basic statistics like assembly size, number of contigs, and N50 were compared via customized python scripts. Benchmarking Universal Single-Copy Orthologs (BUSCO) v2.0 (Simão et al., 2015) was run on all subset assemblies in transcriptome mode to quantify the completeness. A dedicated python script was deployed to identify remaining adapter sequences in contigs via BLASTn (Altschul et al., 1990) (*e*-value < 0.01 and word_size = 4) and to clip them afterwards. Assembled sequences were kept if the surviving part still exceeds the minimal length cutoff. A dedicated python script was deployed to distinguish true plant contigs from bacterial and other contaminations. First, BLASTn (Altschul et al., 1990) with stringent parameters (*e*-value < 0.00001, alignment length >100, similarity >80%) against the *Jatropha curcas* GZQX0401 genome sequence (GCA_000696525.1; Zhang et al., 2014) was applied. Second, all contigs without a significant hit were subjected to a subsequent BLASTn (Altschul et al., 1990) against nt. All sequences with best hits against bacterial genomes were removed, while sequences without hits were kept in this step. Finally, sequences shorter than 400 nt were removed from the final assembly. Since the assembly is based on 2 × 250 nt PE reads, smaller contigs are probably assembly artifacts.

### Prediction of encoded peptides

Peptide-encoding contigs were identified by a python script, based on results from Transdecoder (Haas et al., 2013), ORFfinder (Wheeler et al., 2003), and ORFpredictor (Min et al., 2005). Local alignments for all predicted peptide sequences against Swiss-Prot (Bairoch and Apweiler, 2000; The UniProt Consortium, 2017) were computed via DIAMOND (Buchfink et al., 2014). The longest continuous peptide sequence per contig starting with Methionine was selected unless similarity to a Swiss-Prot sequence pointed toward another predicted peptide sequence on the same contig. In case of multiple predicted sequences with similarity to Swiss-Prot sequences, the selection was based on the alignment score. Finally, only predicted sequences longer than 100 amino acids or with a significant sequence similarity to a Swiss-Prot entry (*e*-value < e-10) were kept.

### Annotation transfer

Reciprocal best BLAST hits (RBHs) against *Arabidopsis thaliana* Araport11 representative peptides (Cheng et al., 2017) were identified as previously described (Pucker et al., 2016) to transfer the functional annotation. In addition, RBHs against *Beta vulgaris* BeetSet-2 (Minoche et al., 2015), *J. curcas* JatCur_1.0 (Zhang et al., 2014), and *Vitis vinifera* PN40024 with the annotation V2.1 provided by CRIBI (Vitulo et al., 2014) were identified to cover a broad range of phylogenetically diverse plant species. All predicted peptide sequences were screened by InterProScan5 (Finn et al., 2017) to assign GO terms.

### Transcript abundance quantification

Reads from tissue-specific data sets were mapped to the final transcriptome assembly via STAR (Dobin et al., 2013) requiring at least 90% of the read sequence to match with at least 95% identity. featureCounts (Liao et al., 2014) was applied to quantify the abundance of all sequences in the assembly. Since most transcripts were represented by multiple contigs probably due to different splice variants and alleles, we decided to include multi-mapped reads. VENN diagram generation was performed at http://bioinformatics.psb.ugent.be/webtools/Venn/ to illustrate tissue-specific transcript abundance.

## Results

**Figure 1:**
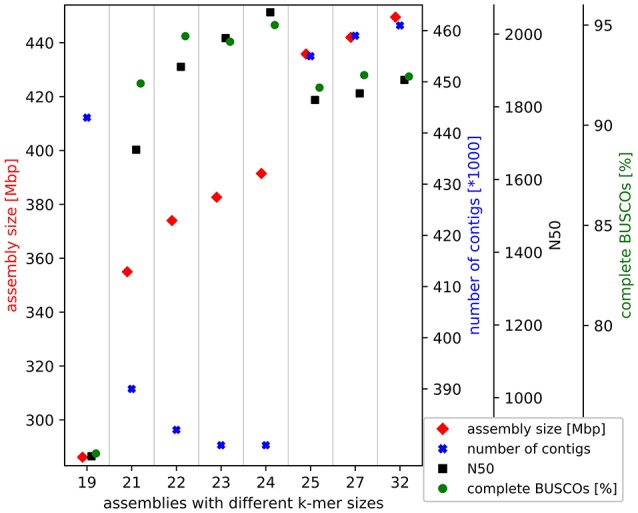
*Croton tiglium de novo* transcriptome assembly benchmarking. Different k-mer sizes were tested for this *de novo* transcriptome assembly via Trinity (Grabherr et al., 2011). Results are evaluated based on assembly size, continuity and recovered benchmarking genes (BUSCOs).

In total, 45.5 million 2 × 250 nt paired-end reads were assembled into the 391.5 Mbp transcriptome comprising 379,585 contigs. Through all filter steps the size was reduced to 345.7 Mbp comprising only 224,425 contigs. This Transcriptome Shotgun Assembly project has been deposited at DDBJ/EMBL/GenBank under the accession GGDV00000000. The version described in this paper is the first version, GGDV01000000. The high continuity of the assembled contigs can be described by the E90N50 of 3,115 nt and the E90N90 of 1,645 nt. These statistics are Nx values of an assembly subset, which accounts for 90% of the expression. The completeness check indicated a sufficient amount of sequencing data was generated. An assembly with only 20% of the sequencing data almost reached the final assembly in terms of size and contained genes. The selected k-mer size of 24 for the final assembly resulted in the best assembly based on size, continuity and recovered benchmarking genes (Figure 1). Benchmarking Universal Single-Copy Orthologs (BUSCO) revealed the presence of 95.1% complete BUSCO genes in the initial assembly. In addition, 2.6% of all BUSCO genes are present in fragmented form and only 2.3% are missing in this *de novo* transcriptome assembly.

A set of 122,206 representative peptide sequences (Supplementary File 1) was inferred by selecting only the best predicted peptide sequence per contig (see methods for details). The position of the corresponding protein coding sequence was identified (Supplementary File 2). Comparison of these predicted peptide sequence to peptide sequence sets of well annotated plant species like *A. thaliana* resulted in 8,858 RBHs. These hits enabled the transfer of functional annotation information in addition to 113,097 assigned GOs (Supplementary Table 2). Comparison to additional plant genomes revealed 8,623 RBHs against *B. vulgaris*, 10,822 against *J. curcas*, and 9,687 against *Vitis vinifera*.

**Figure 2:**
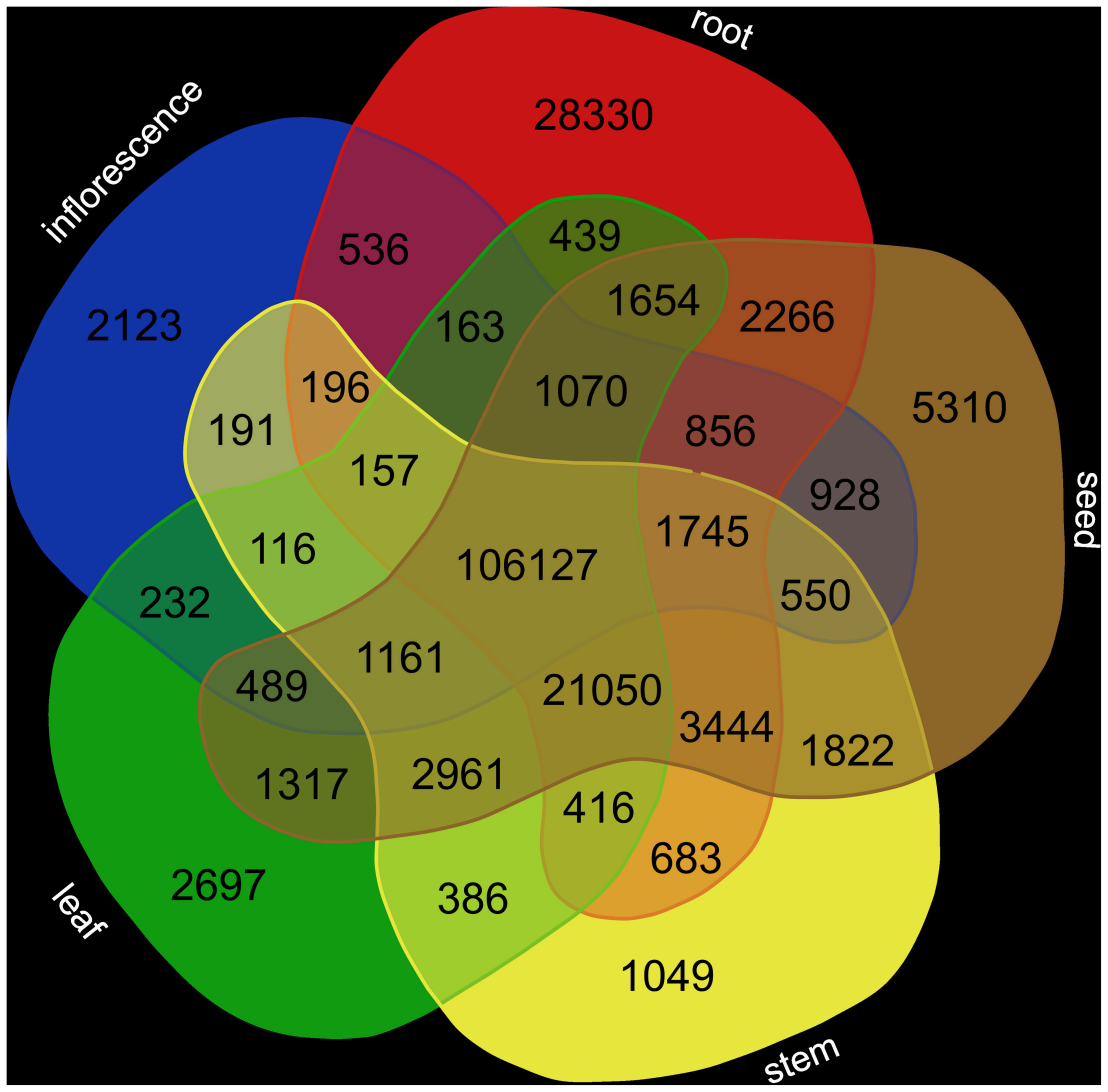
Transcript abundance in different tissues. Transcript abundance was quantified based on RNA-Seq read mapping via STAR (Dobin et al., 2013) and following summarization via featureCounts (Liao et al., 2014).

Analysis of the tissue-specific abundance of the corresponding transcripts (Supplementary Table 3) revealed a high number of shared transcripts (Figure 2). The number of tissue-specific transcripts ranges from 1,049 (stem) to 28,330 (root).

## Discussion

The transcriptome sequences of *C. tiglium* were assembled *de novo* and tissue-specific abundance of transcripts was quantified. High quality of this *de novo* transcriptome assembly is indicated by the high percentage of completely recovered BUSCO genes, since other recent *de novo* transcriptome assemblies of plants reached slightly lower values of up to 93% (Babineau et al., 2017; Blande et al., 2017). Sufficient sequencing depth is indicated by the number of detected complete BUSCO genes in subset assemblies. Probably, most genes would be represented in a transcriptome assembly, based on only 20% of the provided data set. A high number of assembled alternative transcripts per gene could explain some of the reported duplicated BUSCOs. A strong heterozygosity of *C. tiglium* would be another explanation, because both alleles could be represented. Not all assembled transcript sequences were identified in samples of single tissues. One explanation is the nature of the normalized library used for the generation of the *de novo* assembly leading to an enrichment of rare transcripts to enable a comprehensive representation of the transcriptome. In contrast, tissue-specific data sets are dominated by abundant transcripts and thus do not recover all transcript in this assembly.

This *de novo* transcriptome assembly can serve as a reference for the investigation of over 1,200 species in the large genus Croton. The availability of comprehensive sequence information for *C. tiglium* is the first step toward the development of medical applications. Enzymes for the synthesis of various secondary metabolites described in this species may be identified from the provided set of predicted peptides, assembled transcripts, or even based on the raw sequencing reads.

Efforts were already made toward the identification of genes putatively involved in the biosynthesis of the guanosine analog isoguanosine. The transcriptome resource of *C. tiglium* revealed an additional isoform of the *GMPS* gene involved in GMP synthesis (Supplementary File 3). Heterologous expression and *in vitro* enzyme assays of the purified enzymes followed by HPLC/MS analysis revealed substantial differences in the reaction products of the two isoforms that differ from a GMP standard, supporting the postulated involvement of this additional isoform in the isoguanosine biosynthesis (Karsten et al., 2017).

## Direct link to deposited data

All raw sequencing reads were submitted to the SRA https://trace.ncbi.nlm.nih.gov/Traces/sra/sra.cgi?study=SRP123217. The *de novo* assembled transcript sequences are available at DDBJ/EMBL/GenBank through the BioProject https://www.ncbi.nlm.nih.gov/bioproject/416498.

## Author contributions

MH, SV, and WK harvested the samples and extracted the RNA. BP performed bioinformatic analysis of the sequencing data. MH, JD, JK, CR, and BP wrote the manuscript.

### Conflict of interest statement

The authors declare that the research was conducted in the absence of any commercial or financial relationships that could be construed as a potential conflict of interest.
